# Insights into the Mechanism Underlying the Symbiosis between Seagrass and a Lulworthiaceae Fungus

**DOI:** 10.1007/s00248-026-02808-0

**Published:** 2026-07-10

**Authors:** Xiao Wang, Jiawei Chen, Yang Tang, Hongbin Liu

**Affiliations:** 1 Department of Ocean Science, The Hong Kong University of Science and Technology, Kowloon, Hong Kong; 2 Department of Food Science and Nutrition, Faculty of Science, The Hong Kong Polytechnic University, Kowloon, Hong Kong; 3 Department of Infectious Diseases and Public Health, Jockey Club College of Veterinary Medicine and Life Sciences, City University of Hong Kong, Kowloon, Hong Kong

**Keywords:** Lulworthiaceae fungi, Seagrass, Plant-fungal interaction, Marine fungi, Plant growth-promoting activity

## Abstract

Various fungi have been identified in seagrass compartments; however, the exact nature of their interactions with these marine flowering plants remains largely uncharacterized. The partnership between seagrasses and Lulworthiaceae fungi represents a typical and compelling example of such an underwater association. Here, we combine UHPLC-MS/MS, transcriptomics, and plant growth assays to validate the putative symbiosis between the seagrass *Halophila ovalis* and the Lulworthiaceae fungus *Halophilomyces hongkongensis*, and elucidate the underlying mechanism of this relationship. We confirmed that *H. hongkongensis* produces the phytohormone indole-3-acetic acid (IAA). Through transcriptomic analysis, we proposed its IAA biosynthesis pathways. All identified IAA biosynthesis genes were upregulated in seagrass roots/rhizomes compared to rhizosphere sediments, with the tryptophan aminotransferase gene (indole-3-pyruvic acid pathway) exhibiting a significant increase. Furthermore, *H. hongkongensis* was confirmed as a plant growth-promoting fungus; its culture filtrates promoted *Arabidopsis thaliana* shoot and lateral root/root hair growth, an effect strongly correlated with IAA production and highly likely in seagrass. Beyond IAA-specific analyses, its upregulated genes were significantly enriched in pathways such as tryptophan metabolism, starch/sucrose metabolism, and DNA replication. Collectively, these results indicate that *H. hongkongensis* establishes a growth-promoting symbiosis with *H. ovalis* by upregulating its IAA biosynthesis genes and secreting IAA; in return, the host provides carbohydrates that sustain fungal metabolism and support active DNA replication. This study provides the first mechanistic verification of a seagrass-Lulworthiaceae symbiosis, significantly advancing our understanding of marine plant-fungal interactions. It also demonstrates the first IAA-linked plant growth-promoting capacity of a member from the cryptic marine fungal family Lulworthiaceae.

**Supplementary Information:**

The online version contains supplementary material available at https://doi.org/10.1007/s00248-026-02808-0.

## Introduction

Plants associate with diverse fungi that inhabit their internal tissues and surrounding environments. These plant-associated fungi have received considerable attention due to their profound impact on plant health, fitness, and their role in nutrient cycling [1, 2]. Plant-fungal interactions range from valuable to harmful associations, which have been widely acknowledged in terrestrial ecosystems. For instance, over 80% land plants establish symbiotic relationships with mycorrhizal fungi. These symbionts exclusively colonize plant roots and extend into the rhizosphere, facilitating nutrient and water uptake for the host in exchange for photosynthetic carbon [2, 3]. Fungal endophytes that colonize the inner plant tissues asymptotically (i.e., without causing apparent disease symptoms in the host) [1] are also capable of establishing symbiotic relationships with plants [4]. Unlike mycorrhizal fungi, endophytes may reside not only in roots but also in stems/rhizomes or leaves. As facultative symbionts, their development does not strictly rely on the host, allowing them to be cultured on artificial media [5, 6]. Endophytic fungi have the potential to confer benefits to their host plants, such as promoting growth [7] and enhancing tolerance to biotic and abiotic stresses [5, 8]. Conversely, plant-associated fungi could also be pathogenic, leading to disease and the eventual death of plants [9].

In contrast to the extensive studies in terrestrial ecosystems, the plant-fungal interactions remain poorly understood in marine environments. Seagrasses, the only flowering plants fully adapted to a submerged marine lifestyle, associate with fungi that have been relatively overlooked [10]. Consequently, existing studies on seagrass-associated fungi are largely limited to isolation, taxonomy [11–13] and community structure [14, 15], with little investigation into how exactly these fungi interact with the seagrass. Only a few notable exceptions exist; for example, a recent study demonstrated the potential contribution of fungal endophytes to the defense of the seagrass *Thalassia testudinum* against pathogenic *Labyrinthula* [16]. Another study suggested a saprobic role for the seagrass *Posidonia oceanica* mycobiont, which progressively degrades the seagrass from living organs to the matte or litter [17]. Beyond these cases, however, the beneficial or detrimental effects of the majority of fungi on seagrasses are generally unknown [10]. A few hypotheses have been proposed [18–20], such as dark septate fungi that grow on the root surface of the Indo-Pacific seagrass *Thalassodendron ciliatum* may offer biotic stress protection and aid in nutrient accumulation/stabilization [20]. While intriguing, these hypotheses remain vague, as they were inferred only from microscopic observations, leaving the exact nature of these putative symbioses unclear.

The family Lulworthiaceae is the sole family in the order Lulworthiales. It is characterized by prevalence in environments subject to constant or periodic immersion by seawater, typically within intertidal and subtidal zones. The members are frequent inhabitants of seagrass tissues across various species, including recent discoveries in *P. oceanica* [21], *Halophila ovalis* [22], and *Thalassodendron ciliatum* [11]. Other substrates, such as submerged/drifted wood and seaweeds [21, 23], also represent hotspots for colonization of Lulworthiaceae fungi. Within the context of generally enigmatic seagrass-fungal interactions, the example between seagrasses and Lulworthiaceae fungi presents a typical and thought-provoking case.

Among the Lulworthiaceae fungi hosted by seagrasses, *Halophilomyces hongkongensis* was initially described as a novel species in a new genus in 2024 [22]. It is a slow-growing fungus and was repeatedly isolated from surface-sterilized roots and rhizomes of the seagrass *H. ovalis* in an intertidal zone of Hong Kong. We subsequently employed Illumina amplicon sequencing to compare the fungal communities across multiple *H. ovalis* inner and surrounding compartments over a period of one year. The results further emphasized the uniqueness of *H. hongkongensis*, identifying it as the only fungus that dominates and enriches the narrow fungal spectrum of *H. ovalis* roots/rhizomes [24]. Based on these findings, we determined a stable and strong relationship between *H. ovalis* and *H. hongkongensis*, which led us to hypothesize a possible symbiosis between the two [24]. These works provided an essential foundation for the present study, which aims to validate the nature of this symbiotic relationship.

Globally, around 70 seagrass species have been reported, distributed in five families [25]. *H. ovalis* (commonly known as spoon seagrass) belongs to the family Hydrocharitaceae and is considered the most widely distributed seagrass in Hong Kong, where it is found alongside four other species: *H. beccarii* and *H. minor* from the same genus, as well as *Zostera japonica* (Zosteraceae) and *Ruppia maritima* (Ruppiaceae) (https://www.hongkongseagrass.com). This species is also broadly distributed in other tropical, subtropical, and temperate regions, being particularly common in the Tropical Indo-Pacific [26]. Given the significant ecological services provided by seagrass ecosystems, including supporting biodiversity, protecting shorelines, and contributing to carbon sequestration [27], understanding the functional role of their fungal partners is critical.

The objective of this study is to verify our hypothesis of the seagrass-fungal symbiosis between *H. ovalis* and the Lulworthiaceae fungus *H. hongkongensis*, to elucidate the mechanism of their interaction. We used UHPLC-MS/MS to identify metabolites of *H. hongkongensis*, confirming its ability to produce the plant hormone indole-3-acetic acid (IAA). We further employed transcriptomics to resolve the IAA biosynthesis pathways/genes of *H. hongkongensis*, and to determine the activity and expression levels of these relevant genes in field-collected *H. ovalis* roots and rhizomes, as well as rhizosphere sediments. Finally, we assessed the in vitro plant growth-promoting activity of this fungus.

## Materials and Methods

### Fungal Materials

The two *H. hongkongensis* strains used in this study were originally isolated from the rhizome (HOMAR4) and root (HOMAR10) of the seagrass *H. ovalis* in an intertidal zone of Tung Chung Bay, Lantau Island of Hong Kong [22]. These strains were transferred from Potato Dextrose Agar (PDA) slant media, which had been preserved at 4 °C in our laboratory at The Hong Kong University of Science and Technology, to new PDA Petri dishes for further use.

## Metabolite Analysis of *H. hongkongensis* by UHPLC-MS/MS

One 7 mm diameter agar cylinder from the outer edge of 14-day-old *H. hongkongensis* strains HOMAR4 and HOMAR10 that had previously grown on PDA media was inoculated into 100 mL of Potato Dextrose Broth (PDB) in 250 mL flasks for the preparation of fungal filtrates, respectively. The inoculated broth was incubated in a shaking incubator at 25 °C, 120 rpm, in the dark for 15 days. After incubation, the mycelia were removed from the broth by filtration through filter paper. The fungal filtrate content was subsequently extracted in methanol; the freeze-dried material was soaked, ground, and sonicated for 10 min in an ultrasonic bath. After centrifugation at 12,000 rpm for 10 min, the supernatant was collected and filtered through a 0.22 μm PTFE membrane for analysis.

The analytical measurements were conducted with a Vanquish UHPLC system coupled with a Q-Exactive HF mass spectrometer (Thermo Fisher Scientific, Seattle, Washington, USA). Chromatographic separation of samples was performed at 30 °C using a C18 column (Zorbax Eclipse C18; 1.8 μm; 2.1 × 100 mm), with a mobile phase of 0.1% formic acid solution as solvent A and pure acetonitrile as solvent B, following the gradient: 5% B, 0–2 min; 5%–30% B, 2–6 min; 30% B, 6–7 min; 30%–78% B, 7–12 min; 78% B, 12–14 min; 78%–95% B, 14–17 min, 95% B, 17–20 min; 95%–5% B, 20–21 min; and 5% B, 21–25 min. The flow rate of the mobile phase was 0.3 mL/min, and the injection volume was 2 µL. The Q-Exactive HF mass spectrometer was operated under both electrospray ionization (ESI) positive mode and negative mode. The parameters for both modes are described as follows: electrospray voltage, 3.5 kV; sheath gas flow, 45 arb; auxiliary gas flow, 15 arb; sweep gas flow rate, 1 arb; capillary temperature, 330 °C. Full scan (*m*/*z* 100 ~ 1500) and data-dependent mass spectrometry (dd-MS2) were used at a resolution of 120,000 (MS1) and 60,000 (MS2), respectively. The collision mode was high energy collision dissociation (HCD).

### RNA Extraction of *H. hongkongensis*, *H. ovalis* Tissues and Rhizosphere Sediments

For the fungal RNA extraction, mycelia were harvested from the outer edge of a 20-day-old *H. hongkongensis* strain HOMAR4 on PDA and transferred into 500 µL of autoclaved phosphate-buffered saline (PBS) solution (0.01 M, pH 7.4) with zirconia beads. The mixture was homogenized for 30 s at 60 Hz using a high-throughput tissue grinder (SCIENTZ-24, SCIENTZ, Ningbo, China), then inoculated into 20 mL of PDB in a 50 mL flask. The PDB was prepared using either Milli-Q water (Culture 1) or seawater (Culture 2). The used seawater was collected from the seagrass area with a salinity of 25 ppt. After 10-day incubation (25 °C, 120 rpm, in the dark), the fungal structure was filtered by Miracloth, washed with PBS, then dried and collected for RNA extraction using RNeasy Plus Plant Kit (QIAGEN). Liquid nitrogen was used to grind the fungal structure.

The *H. ovalis* seagrass samples for RNA extraction were collected on 08 May and 21 August 2024, during the monsoon season of Hong Kong, characterized by frequent rainfall, higher humidity, and temperatures. The *H. ovalis* seagrass is known to be small-sized, with tiny leaves and thin roots/rhizomes; therefore, no apparently observable differences were detected among the collected seagrasses. The collected samples were separated into the below-ground tissues (i.e., roots and rhizomes) and rhizosphere sediment. The seagrass leaves were first removed in the field, then the below-ground seagrass tissues with adhering sediment were collected in 50 mL Falcon tubes and transported to the laboratory. Autoclaved PBS was added to the tube, vortexed for 5 min to release the rhizosphere sediment in the PBS solution. The tissues were picked out and rapidly washed with sterile water. The remaining suspension in the tube was centrifuged at 6000×*g* for 15 min to pellet the sediment, and the supernatant was removed. For seagrass tissue RNA extraction, the samples were first homogenized using liquid nitrogen. Total RNA was extracted with 1 mL of TRIzol Reagent, incubated for 10 min, followed by the addition of 200 µL of chloroform. After vortexing and incubation for 10 min, the mixture was centrifuged at 12,000×*g* for 15 min at 4 °C [28]. The supernatant was collected, and further steps were taken according to the manufacturer’s protocol of the RNeasy Plus Mini Kit (QIAGEN). The rhizosphere sediment RNA was extracted using RNeasy PowerSoil Total RNA Kit (QIAGEN), following the manufacturer’s protocol.

## Transcriptomic Analysis

To obtain the reference transcriptome of *H. hongkongensis*, the total RNA extracted from *H. hongkongensis* HOMAR4 cultures (both Culture1 and Culture2) was used for Poly(A) RNA sequencing (RNA-seq), in which qualified RNA samples were first subjected to poly(A) mRNA isolation using the NEBNext Poly(A) mRNA Magnetic Isolation Module (NEB) and then used for library generation. To obtain the transcriptional profiles of all organisms (only *H. hongkongensis* were focused for further analysis), the total RNA extracted from below-ground tissues (four and six samples from May and August, respectively) and rhizosphere sediment (from May) was used for metatranscriptomic (metaT) sequencing, in which qualified RNA samples were first subjected to rRNA removal using a Ribo-Zero Plus rRNA Depletion Kit (Illumina) and then used for library generation with a NEBNext Ultra RNA Library Prep Kit for Illumina (NEB). Next-generation sequencing (NGS) was performed using a NovaSeq 6000 system (Illumina), generating 150-bp paired-end reads. Each sample was sequenced to generate approximately 10 Gb of data.

Raw RNA-seq data from fungal cultures 1 and 2 were trimmed using Trimmomatic v0.39 [29] and then co-assembled using Trinity v2.14.0 [30]. Similar transcripts were clustered using CD-HIT [31] with ‘-c 0.95’ parameter, and a single representative transcript was retained for each group. The longest open reading frame (ORF) of fungal transcripts was predicted using TransDecoder v5.5.0 (github.com/TransDecoder/TransDecoder) and annotated against various databases, including the NCBI non-redundant protein (NR) database [32], Kyoto Encyclopedia of Genes and Genomes (KEGG) database [33], the universal protein knowledgebase (UniProtKB) [34], the carbohydrate-active enzymes database (CAZy) [35], and the transporter classification database (TCDB) [36] using diamond v2.1.6 [37] with parameters ‘--sensitive -k 1 -e 1e-20 --id 30 --query-cover 75 --subject-cover 75’. To reveal the transcriptional activities of ORFs, raw metaT data were first trimmed using Trimmomatic v0.39 [29]. The clean reads of metaT samples were aligned to ORFs predicted from the *H. hongkongensis* RNA-seq assembly using CoverM v0.7.0 [38] in ‘contig’ mode with parameters ‘--methods TPM --min-read-percent-identity 95 --min-read-aligned-percent 75’. The RNA-seq data from fungal cultures were also mapped using the same pipeline. The proportion of reads generated from RNA-seq or metaT from each sample annotated as *H. hongkongensis* is reported in Table S1.

## Plant Growth Assay

To verify *H. hongkongensis* as a plant growth-promoting fungus, we evaluated the influence of its culture filtrates on seedling growth of *Arabidopsis thaliana* (ecotype Col-0) by transferring the *A. thaliana* seedlings onto 1/2 Murashige and Skoog (MS) medium with or without the supplementation of *H. hongkongensis* culture filtrates and comparing the plant growth traits after incubation.

For the preparation of fungal filtrates, three 5 mm diameter agar cylinders from the outer edge of the *H. hongkongensis* strain HOMAR4 on PDA were inoculated into three types of liquid media, respectively: PDB, PDB supplemented with 0.1 mg/mL L-tryptophan (PDB_Trp), and Czapek Dox Broth (CDB) supplemented with 0.1 mg/mL L-tryptophan (CDB_Trp). Fifty milliliters of each medium and fungal inocula in 100 mL flasks were incubated at 25 °C, 120 rpm, in the dark for 15 days. Each culture was then filtered through a 0.22 μm PES membrane to separate the fungal structure from the liquid phase (filtrate), which was further mixed with autoclaved 1/2 MS medium in a ratio of 1:9 and poured into Petri dishes [39]. The composition of all culture media is reported in Table S2.

To obtain the *A. thaliana* seedlings, the sterilized seeds were planted on 1/2 MS medium, and placed nearly vertically in controlled conditions: 12 h photoperiod, light intensity 300 µmol/m^2^/s, temperature 21 °C. The seeds were surface sterilized with 75% ethanol for 1 min, 3% NaClO for 15 min, and washed with sterile water. Then, the seeds were sown on 1/2 MS plates sealed with microporous and breathable tape [40], and placed under low temperature at 4 °C for 2 days before being transferred to controlled conditions.

Seven-day-old *A. thaliana* seedlings with similar growth conditions were carefully picked and transferred to the above-mentioned 1/2 MS media with different fungal filtrates (PDB, PDB_Trp, and CDB_Trp), also to 1/2 MS without supplementation of fungal filtrate as a control. After further growth for 11 days, the *A. thaliana* growth traits (including shoot fresh weight and leaf area, root fresh weight and primary root length) were recorded, and the plates were photographed. Leaf area and primary root length were determined using ImageJ (version 1.54 g) software.

### Statistical Analysis

All the statistical analyses were performed using R version 4.3.2. Differential expression analysis of genes between compartments (seagrass below-ground tissue *versus* rhizosphere sediment) was performed using the edgeR package v.3.30.3 [41]. Only genes with a *P*-value < 0.01 and |Log_2_(Fold change)| > 1 were regarded as differentially expressed genes (DEGs). KEGG enrichment analysis was implemented using the “enricher” function in the clusterProfiler v.3.16.1 package [42]. In the enrichment analysis of DEGs, only pathways with a *P*-value < 0.05 were regarded as significantly enriched. Differences in plant growth traits between treatments were compared by Student’s t-test.

## Results and Discussion

### IAA Production by *H. hongkongensis*

UHPLC-MS/MS detected IAA among the metabolites produced by *H. hongkongensis* in both HOMAR4 and HOMAR10 culture broths, respectively (Table S3). IAA is a fundamental phytohormone of the auxin class that regulates plant growth and development by inducing cell division and elongation, and it plays a key role in plant-microbe symbiotic interactions [43, 44]. Fungal endophytes of various land plants have been reported to produce IAA, which explains one of the main mechanisms for their growth-promoting effects on hosts [45]. The confirmation of IAA production by *H. hongkongensis* based on UHPLC-MS/MS analysis led us to further investigate its potential role in seagrass growth.

### IAA Biosynthesis Pathways of *H. hongkongensis*

**Fig. 1:**
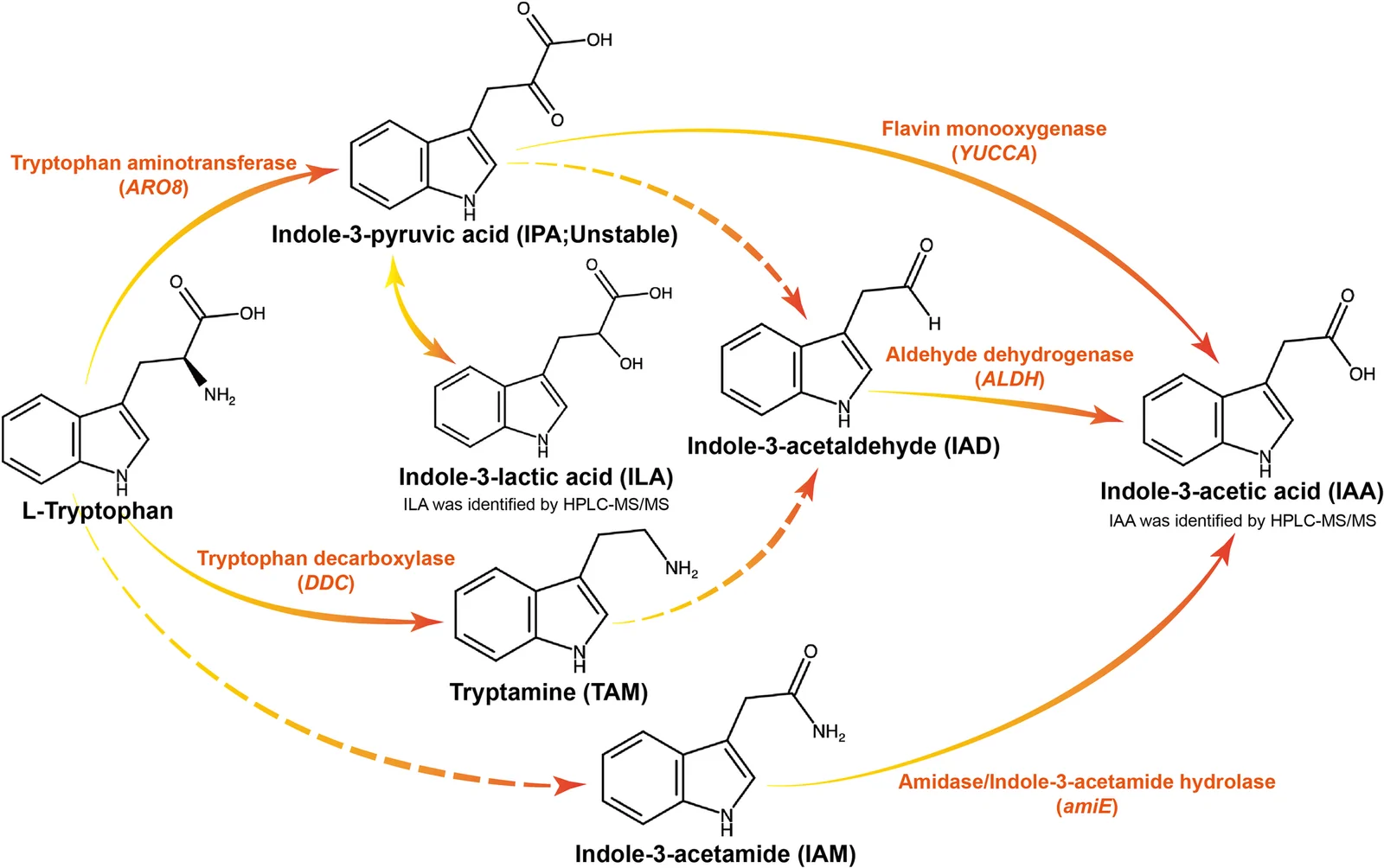
Four proposed tryptophan-dependent indole-3-acetic acid (IAA) biosynthesis pathways for *H. hongkongensis* based on UHPLC-MS/MS and transcriptomic evidence: the indole-3-pyruvic acid (IPA) pathway, tryptamine (TAM) pathway, indole-3-acetamide (IAM) pathway, and YUC pathway. Note that the direct conversion of IPA to IAA (YUC pathway) is common for plants but has not been fully clarified for fungi. IAA and indole-3-lactic acid (ILA) were identified in two liquid cultures of *H. hongkongensis* by UHPLC-MS/MS analysis. IPA is known to be unstable and spontaneously converts into ILA. The genes and encoding enzymes are indicated in each pathway. The solid arrows indicate the corresponding genes for the conversion were detected by transcriptomic analysis, or the metabolite was detected by UHPLC-MS/MS, while dashed arrows are inferred from previously known IAA biosynthesis pathways for fungi

We conducted RNA-seq on *H. hongkongensis* to identify the genetic basis for its IAA production. The annotation results from the *H. hongkongensis* RNA-seq assembly are listed in Table S4. Transcriptomic analysis identified the expression of five genes involved in the IAA biosynthesis process (Fig. 1): tryptophan aminotransferase (*ARO8*), tryptophan decarboxylase (*DDC*), amidase/indole-3-acetamide hydrolase (*amiE*), aldehyde dehydrogenase (*ALDH*), and flavin monooxygenase (*YUCCA*). Among these enzymes, ARO8 and DDC convert L-tryptophan to indole-3-pyruvic acid (IPA) and tryptamine (TAM), respectively. amiE converts indole-3-acetamide (IAM) to IAA. ALDH converts indole-3-acetaldehyde (IAD) to IAA, while YUCCA directly converts IPA to IAA, thereby bypassing the formation of IAD.

Although we did not detect genes for the conversions from IPA and TAM to IAD, as well as from L-tryptophan to IAM, these steps were inferred from previous studies [46–48]. Among the four pathways that we proposed, the IPA pathway (Tryptophan→IPA→IAD→IAA) is considered the predominant route for IAA biosynthesis in fungi [48, 49], while the IAM pathway (Tryptophan→IAM→IAA) is one of the most well-studied pathways. The TAM pathway (Tryptophan→TAM→IAD→IAA) is often regarded as the side route for IAA production and generally coexists with other pathways [48, 50]. The YUC pathway (Tryptophan→IPA→IAA), which contains flavin monooxygenase encoded by the *YUCCA* gene family, however, has primarily been reported for IAA biosynthesis in plants [51]. Although the YUC pathway is not yet fully characterized in fungi, our data revealed transcripts for flavin monooxygenase, indicating the potential activity of this pathway in *H. hongkongensis*. The existence of this pathway in fungi is also supported by other fungal genomic and *YUCCA* protein inhibition studies [52, 53].

In addition to the transcriptomic evidence for the IAA biosynthesis pathways, we also searched the UHPLC-MS/MS results for intermediate compounds (such as IPA, IAD, TAM, IAM) and other relevant compounds, such as indole-3-lactic acid (ILA) and indole-3-ethanol (TOL), as IPA and IAD are unstable and dynamically convert to these compounds, respectively [47, 49]. We detected ILA from the metabolites produced by *H. hongkongensis* (Table S3); therefore, we also added the conversion between IPA and ILA in Fig. 1. Although other compounds were not shown in the metabolite list, they may still be present in the broth but could have gone undetected due to the absence of their reference standards in the UHPLC-MS/MS analysis database.

### Active Expression of *H. hongkongensis* IAA Biosynthesis Genes in Living *H. ovalis *Tissues and Rhizosphere Sediments

By mapping the metaT data from *H. ovalis* below-ground tissues and rhizosphere sediments onto the *H. hongkongensis* reference assembly, we were able to profile the gene expression of *H. hongkongensis* within its natural habitat (Table S5). We found that the five *H. hongkongensis* IAA biosynthesis genes identified in culture experiments were also actively expressed in the field-collected *H. ovalis* below-ground tissues and rhizosphere sediments (Table S6). This confirms that *H. hongkongensis* possesses the capacity to produce IAA not only under experimental cultivation conditions but also in its natural environment. The approach of mapping environmental metaT reads to the RNA-seq assembly of *H. hongkongensis* under culture conditions allowed us to visualize fungal gene expression in the seagrass meadow; however, it may not capture genes expressed exclusively in its natural environment. Although this approach proved sufficient for the specific aim of this study, which is to determine whether IAA biosynthesis genes identified in culture are also expressed in natural seagrass-associated environments, it may have limitations for broader applications.

**Fig. 2:**
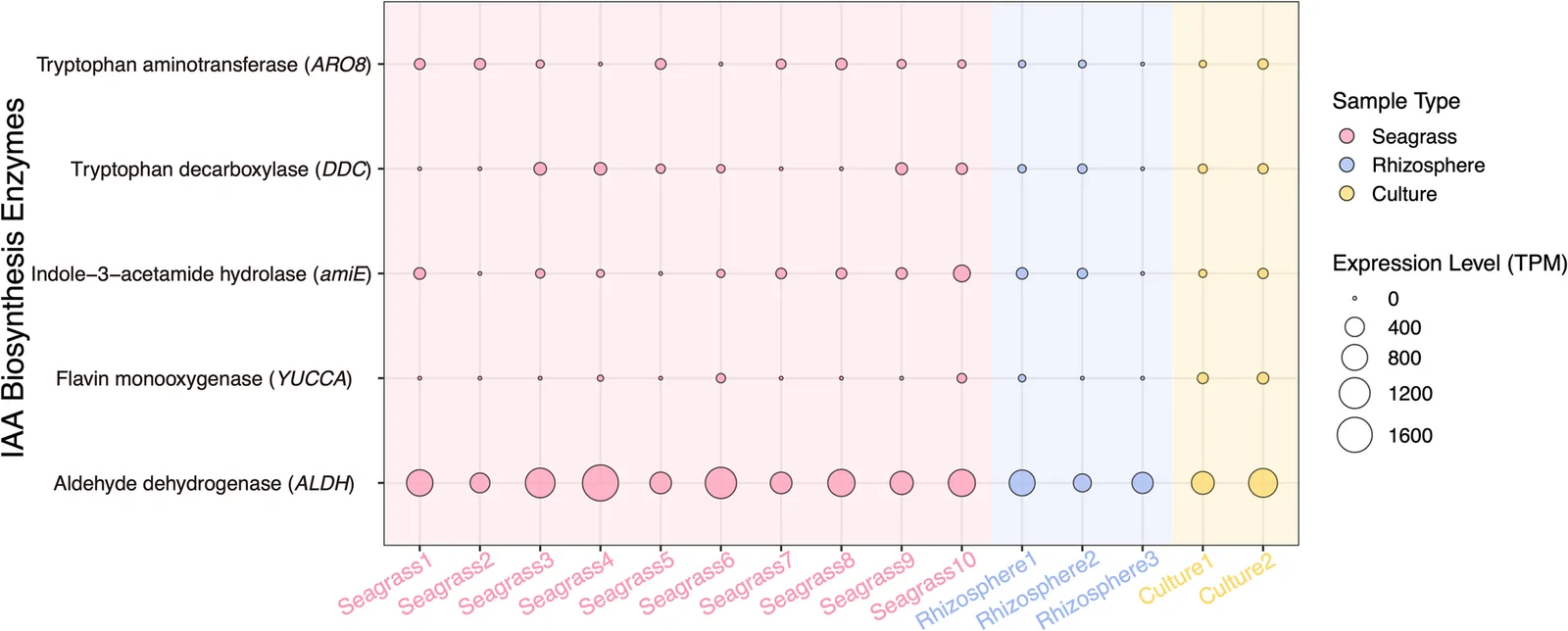
Expression levels of *H. hongkongensis* IAA biosynthesis genes in *H. ovalis* below-ground tissues, rhizosphere sediments, and Potato Dextrose Broth (PDB) cultures. The expression level of genes is indicated by Transcripts Per Million (TPM). The size of the bubbles corresponds to the TPM value

**Fig. 3:**
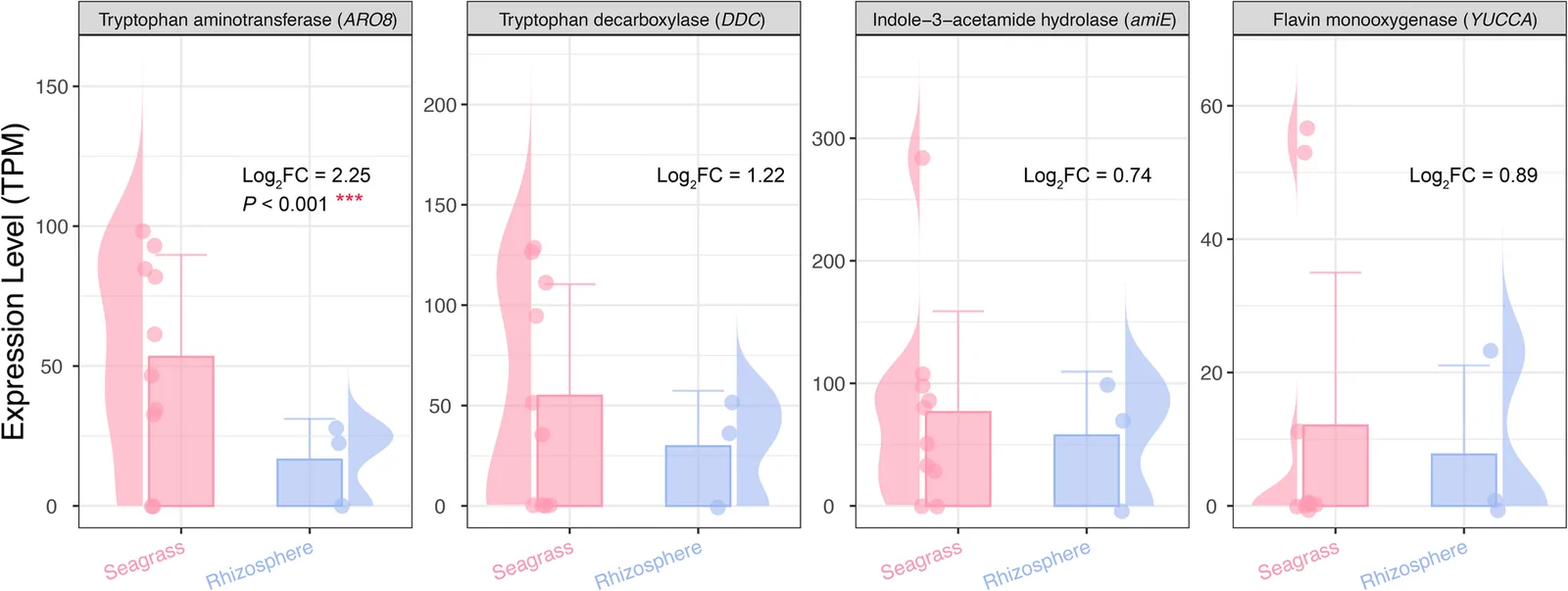
Raincloud plots (combined violin plots, dot plots, and box plots) for comparisons of the expression levels of *H. hongkongensis* IAA biosynthesis genes between seagrass tissues and rhizosphere sediments. Due to the broad metabolic roles of *ALDH*, its upregulation may not specifically indicate involvement in IAA biosynthesis; therefore, it was not included in this figure but is presented in Fig. S1 for clarity. The Log₂(Fold change) (Log₂FC) values from differential expression analysis using the edgeR R package are shown in each plot, with the “***” denoting the single significant result (*P* < 0.001)

The expression levels (transcripts per million, TPM) of these five genes in the *H. ovalis* below-ground tissues, rhizosphere sediments, and cultures are shown in the bubble plot (Fig. 2). Unlike other genes, the expression of *YUCCA* was not detected in a large proportion of seagrass samples, suggesting that the YUC pathway catalyzed by this enzyme may be of minor importance. Furthermore, comparisons of gene expression between seagrass-associated compartments showed that the expression of all five IAA biosynthesis genes was upregulated in seagrass roots/rhizomes compared to the surrounding rhizosphere sediments (Fig. 3 and S1, Table S7). In particular, the upregulation of the gene encoding ARO8, a key enzyme in the IPA pathway, was significant among the five IAA biosynthesis genes (Log₂(Fold change) = 2.25, *P* < 0.001) based on edgeR R package with ‘glm’ method). These results collectively indicate that interaction with the host seagrass triggers upregulation of *H. hongkongensis* IAA biosynthesis genes. The significant upregulation of *ARO8* strongly implicates the IPA pathway as the primary route for IAA production in this symbiotic context, consistent with its established role as the main IAA biosynthesis pathway in fungi.

### Plant Growth-Promoting Effects of *H. hongkongensis*

**Fig. 4:**
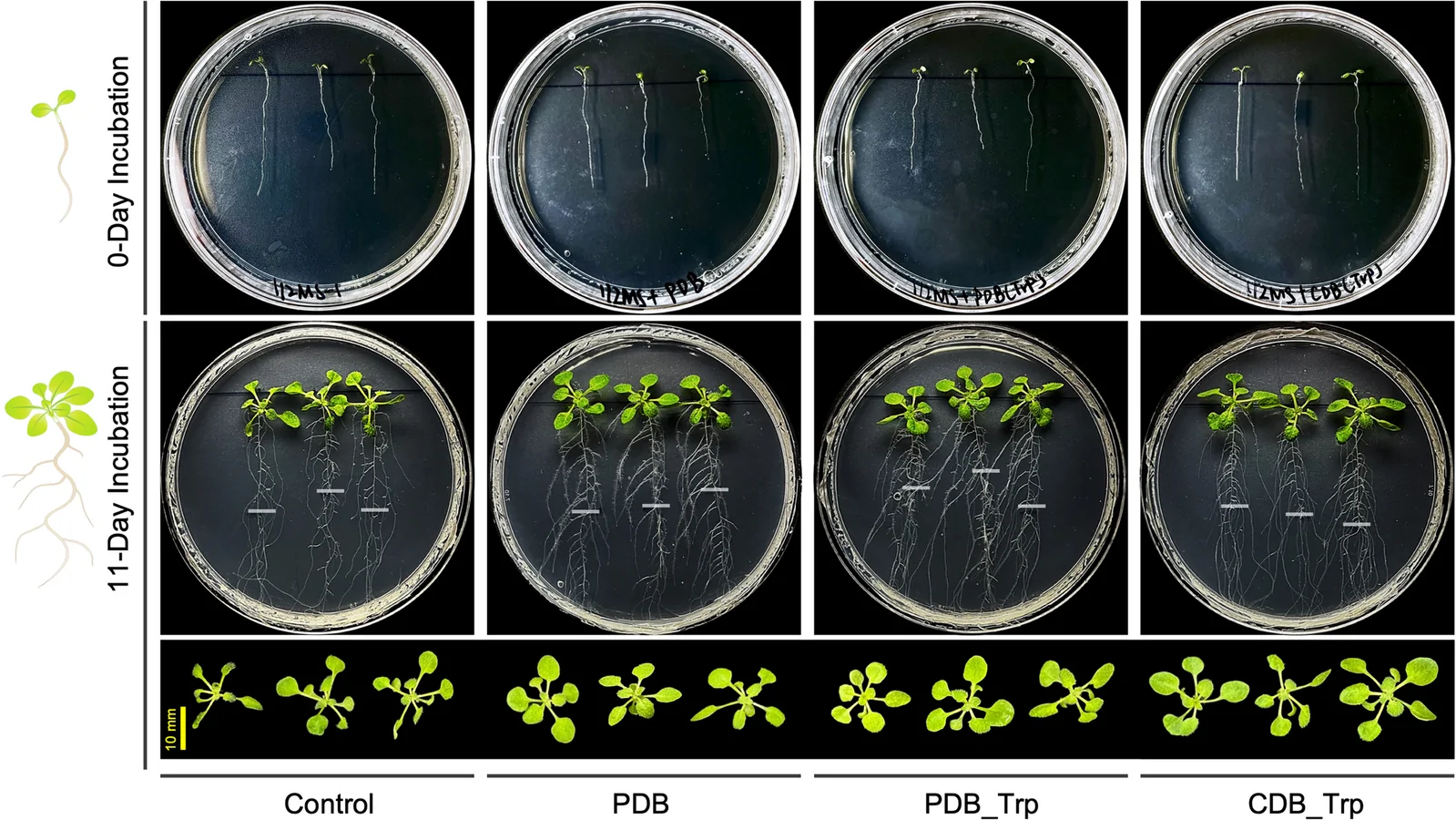
Effects of *Halophilomyces hongkongensis* culture filtrates on the growth of *Arabidopsis thaliana* ecotype Columbia-0. The 7-day-old *A. thaliana* seedlings at the 0-day and 11-day of incubation in the 1/2 Murashige and Skoog (MS) media supplemented with (PDB, PDB_Trp, and CDB_Trp) or without (Control) fungal filtrates. White bars in Petri dishes of 11-day incubation indicate the initial position of the roots at 0-day incubation

The *A. thaliana* seedlings were grown on 1/2 MS media supplemented with different fungal filtrates (PDB, PDB_Trp, and CDB_Trp), and their growth was compared with seedlings grown on basal media (Control). Overall, all three fungal filtrates exhibited positive effects on *A. thaliana* development. Visually, these filtrates promoted *A. thaliana* growth in both shoot and root systems (Fig. 4), resulting in larger leaves and more extensive lateral roots and root hairs compared to the Control.

**Fig. 5:**
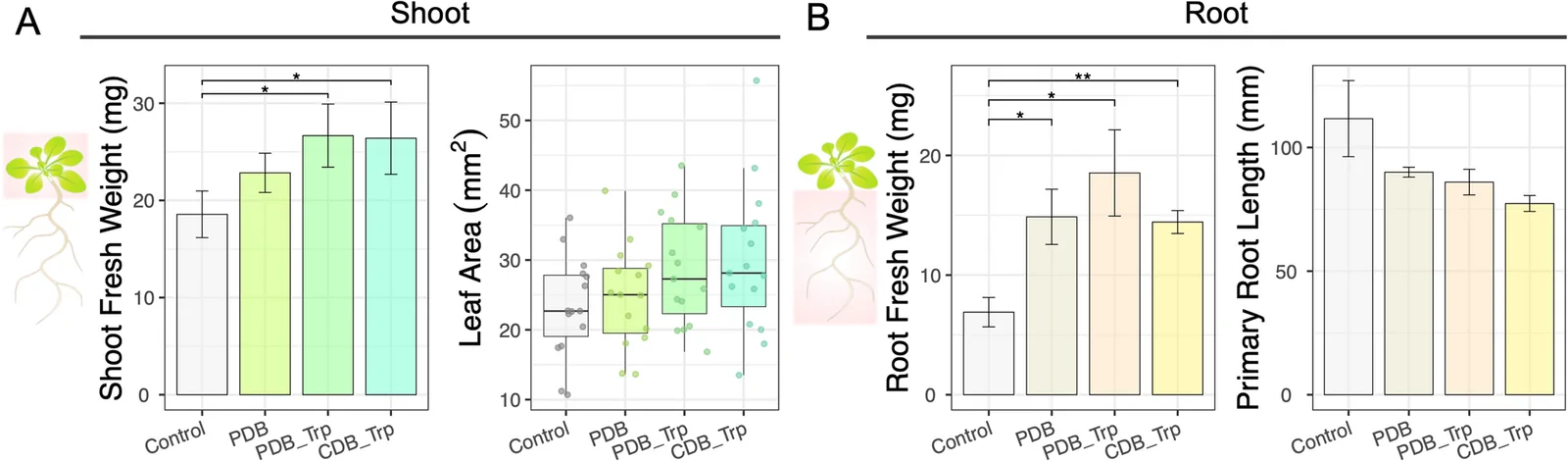
Comparisons of *A. thaliana* (**A**) shoot (shoot fresh weight and leaf area) and (**B**) root (root fresh weight and primary root length) growth traits after 11 days of cultivation. Differences between groups were evaluated by Student’s t-test, indicated as: *P < 0.05, **P < 0.01. For bar plots, data are shown as mean ± SD (*n* = 3). For 1/2 MS media with different fungal filtrates, PDB = fungal filtrates from Potato Dextrose Broth (PDB), PDB_Trp = fungal filtrates from PDB supplemented with L-tryptophan, CDB_Trp = fungal filtrates from Czapek Dox Broth (CDB) supplemented with L-tryptophan

For the shoot system, seedlings treated with any of the three fungal filtrates had higher shoot fresh weight and larger leaf area than the control (Fig. 5A). Filtrates from fungal cultures supplemented with tryptophan (PDB_Trp and CDB_Trp) had a more apparent promoting effect on shoots compared to the PDB filtrate without additional tryptophan. This difference was especially significant for shoot fresh weight (Student’s t-test, *P* < 0.05; Fig. 5A).

For the root system, treatment with fungal filtrates significantly increased root fresh weight (Student’s t-test *P* < 0.05), with seedlings on PDB_Trp medium showing the highest value (Fig. 5B). The increase in root fresh weight was associated with the greater number of lateral roots and root hairs, as observed visually (Fig. 4). In contrast, the primary root length of seedlings in the control treatment was longer than that in any of the fungal filtrate treatments (Fig. 5B). This contrasting effect that promoting root biomass and lateral root formation while inhibiting primary root elongation, is a well-documented and typical response to auxin and has been similarly reported for other endophytic fungi [46, 54–56]. Taken together, the PDB_Trp filtrate had the greatest growth-promoting effect on *A. thaliana*, which is likely attributable to a higher concentration of IAA synthesized from tryptophan precursor in this medium, which contained both supplemental tryptophan and the tryptophan from potato infusion in the PDB.

Overall, the fungal filtrates of *H. hongkongensis* promoted *A. thaliana* growth by enhancing shoot and root fresh weight, as well as leaf area (Figs. 4 and 5), confirming its role as a plant growth-promoting fungus. The mechanism underlying this promotion is strongly linked to its ability to produce the phytohormone IAA, as identified by UHPLC-MS/MS. When combined with the detected higher expression of its IAA biosynthesis genes in seagrass roots/rhizomes compared to rhizosphere sediment, these results collectively indicate that *H. hongkongensis* facilitates seagrass growth, primarily through its IAA production ability.

In this study, the plant growth-promoting ability of *H. hongkongensis* was tested on the model plant *A. thaliana* (Brassicaceae). This plant offers distinct advantages, such as a short life cycle and ease of cultivation/manipulation, making it highly valuable in plant biology studies [57], and a standard organism for assessing fungal endophytes from diverse hosts [46, 58, 59]. The use of the non-host plant *A. thaliana* allowed us to conclusively demonstrate the plant growth-promoting capacity, which is likely applicable to its native seagrass host, *H. ovalis*. Performing growth assays directly on seagrass would be ideal, but this requires established in vitro seagrass cultures. We attempted to culture *H. ovalis* by transferring sterilized explants into MS medium, the most widely used medium for plant culture, with or without *H. hongkongensis* culture filtrates (prepared from liquid culture using Milli-Q water/seawater) or fungal inoculum. The *H. ovalis* were sterilized using 75% ethanol for 2 minutes, and 2% NaClO for 30 s, then the explants were prepared by dissecting the seagrasses from rhizomes, each containing shoots and roots growing from a node, some also with the growing tip (Fig. S2). Despite months of incubation under controlled conditions (25 °C, 100 µmol/m^2^/s light intensity, 12 h/12 h light/dark cycle), no growth was observed. Seagrass tissue culture is technically challenging, with success reported for only a limited number of seagrasses [60–62]. Its success depends on the coordination of multiple aspects, including explant type, efficient sterilization, proper media, hormone combinations, and incubation conditions. Furthermore, unexpected contamination during the process could be common and problematic. The model plant *A. thaliana* has well-established in vitro culture protocols, providing a reliable and efficient system to characterize the plant growth-promoting traits of *H. hongkongensis*.

### Overview of DEGs and Enriched Pathways of *H. hongkongensis* in Seagrass Tissue

Beyond the focused analysis of IAA biosynthesis, we sought to explore other aspects of the interaction by visualizing all DEGs of *H. hongkongensis* in seagrass tissues *versus* rhizosphere sediment in a volcano plot (Fig. S3) and performing KEGG enrichment analysis to identify significantly enriched pathways (Fig. S4).

Enrichment analysis of upregulated genes revealed seven significantly enriched pathways (Fig. S4). Among these, tryptophan metabolism is the pathway encompassing IAA biosynthesis. Enriched starch and sucrose metabolism indicates that the fungus actively utilizes host-derived saccharides, particularly starch and sucrose, which are known to be mainly stored in seagrass below-ground tissues [63]. This may explain the fungus’s preferred colonization of *H. ovalis* below-ground tissues over leaves [22, 24], as these tissues are rich in starch and sucrose. The enrichment of the DNA replication pathway suggests the transition to the seagrass below-ground tissues provides a more favorable niche, triggering enhanced replicative capacity. Significantly downregulated genes were enriched in the carbon metabolism (map01200). DEGs involved in tryptophan metabolism, starch and sucrose metabolism, and DNA replication were highlighted in the volcano plot (Fig. S3).

### The Seagrass-Fungal Beneficial Symbiosis Between *H. ovalis* and *H. hongkongensis*

**Fig. 6:**
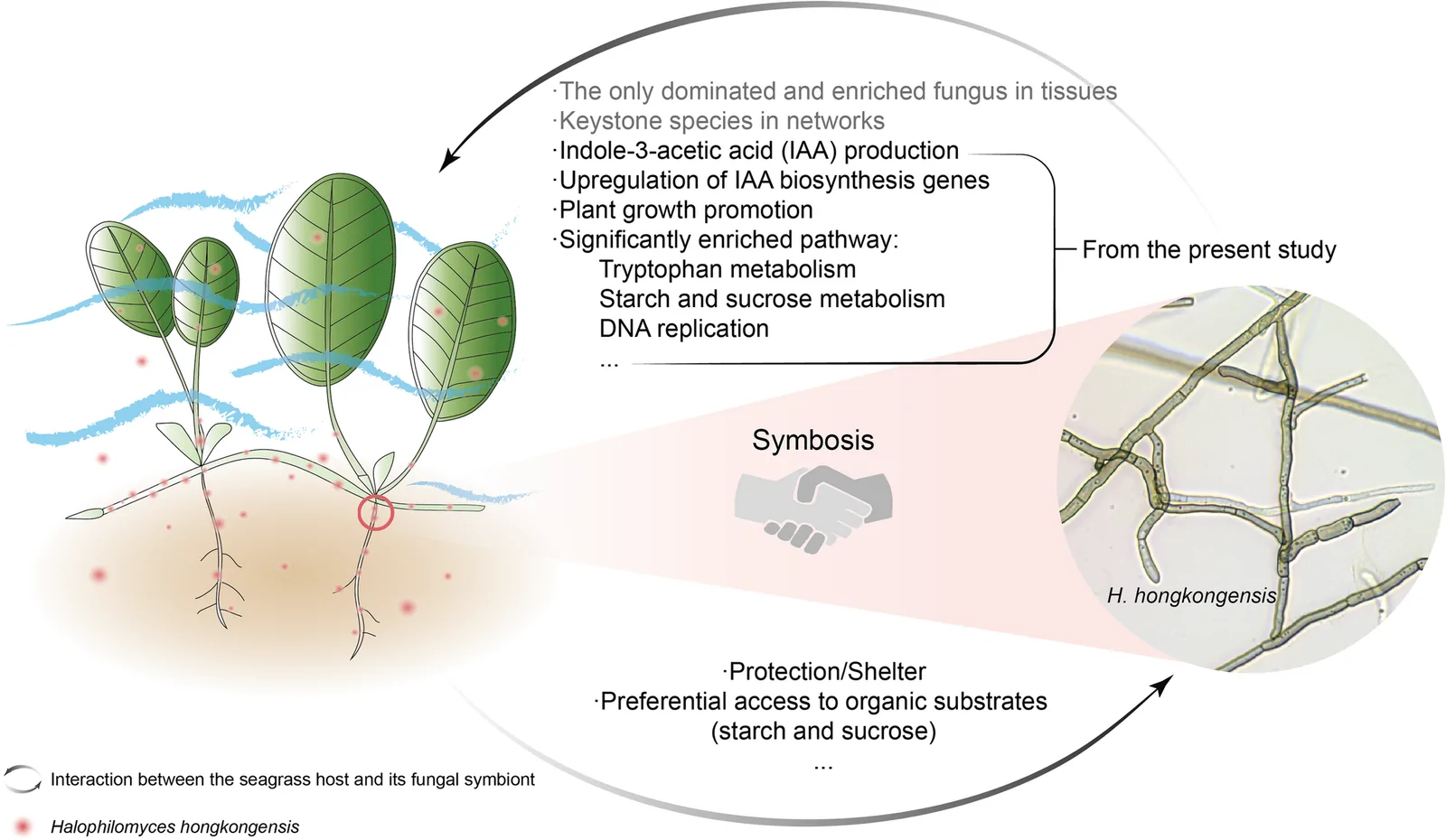
The plant growth-promoting symbiosis between the seagrass *Halophila ovalis* and the Lulworthiaceae fungus *Halophilomyces hongkongensis*. This fungus was initially isolated from *H. ovalis* roots and rhizomes and described as a novel species in a new genus [22]. It dominates the narrow fungal spectra of *H. ovalis* inner tissues, particularly the below-ground roots and rhizomes, is the only fungus that significantly increases in relative abundance inside the seagrass compared to the surrounding compartments, and has been identified as a keystone taxon in fungal-prokaryotic interdomain networks [24]. This study elucidates the mechanistic basis of this symbiotic relationship, as illustrated in the schematic diagram

By integrating UHPLC-MS/MS analysis, transcriptomics, and in vitro plant growth assays, we present complementary evidence for a plant growth-promoting symbiosis between the seagrass *H. ovalis* and the Lulworthiaceae fungus *H. hongkongensis*, a previously undescribed seagrass-fungal relationship. In this symbiotic relationship, *H. hongkongensis* upregulates its IAA biosynthesis genes (especially those of the IPA pathway) and secretes IAA to facilitate seagrass growth, while also actively engaging in DNA replication. In return, the host seagrass provides various benefits to its fungal partner, including protection and preferential access to organic substrates, especially sucrose and starch (Fig. 6) [64, 65].

The elucidation of *H. ovalis*-*H. hongkongensis* beneficial symbiosis was achieved through a multi-faceted approach. It originated from the culture-based isolation and identification of the novel Lulworthiaceae fungus *H. hongkongensis* [22], was followed by Illumina amplicon sequencing to profile its compartmental distribution [24], and was extended in the present study, which employed metabolite profiling, transcriptomic analysis, and in vitro functional assays. While other studies have used the term “symbiosis” to describe the relationship between seagrass and fungi [11, 18, 20], they generally lacked solid evidence for the functional role of the fungal partners. Our work provides the missing functional validation, moving beyond the mere documentation of fungal presence to demonstrate a mechanistic basis for the symbiosis.

Lulworthiaceae fungi have been regarded as lignicolous, and research on this group has been largely confined to taxonomy. Only recently has one study investigated the secondary metabolites of a Lulworthiaceae isolate [66]. The discovery of IAA production by *H. hongkongensis* and its interaction with seagrass expanded our understanding of the ecological lifestyles of Lulworthiaceae, and added functional value to this group of marine fungi. Further investigations should focus on the colonization style of *H. hongkongensis* within host tissues and its role in nutrient cycling of the seagrass ecosystem. Techniques such as fluorescence in situ hybridization and isotopic tracing would be highly valuable in better illustrating this symbiotic relationship.

### Future Perspectives

In the present study, we identified metabolites produced by *H. hongkongensis*, determined and compared the activity and expression levels of fungal genes (especially IAA-related), and finally verified that *H. hongkongensis* promotes plant growth.

Regarding our in vitro plant growth assay, fungal filtrates (filtered fungal broth containing metabolites) promoted *A. thaliana* growth. This protocol, which involves comparing plant growth on basal medium with or without 10% fungal filtrates, was adapted from previous studies [39, 67]. Future studies may consider the potential confounding effects of residual medium-derived nutrients in the fungal filtrates on the studied parameters, though these nutrients were presumably mostly consumed during the long-time fungal culture and are therefore unlikely to substantially influence the results. During trials (data not shown), we observed similar promotion effects, especially for lateral root development, using alternative protocols, including direct co-cultivation of *A. thaliana* with fungal inocula and injection of 50 µL of fungal filtrates into bored holes in the basal medium (as exemplified in [59]). Thus, the observed plant growth-promoting effect in this assay is attributable to fungal metabolites.

Although the plant growth promotion was examined on a model plant rather than the fungus’s original host, due to the difficulties in culturing seagrass in vitro, there is strong evidence to suggest that this IAA-linked ability is highly likely to also apply to its host. Seagrasses possess auxin signaling genes, such as *IAA*, *AUX*/*IAA*, and *ARF* [68], and not only rely on endogenous auxins for key developmental stages [68], but are also auxin-responsive and can benefit from IAA and other auxins [69–71]. Nevertheless, direct evidence for *H. hongkongensis*- or IAA-mediated growth promotion on *H. ovalis* requires further exploration. Promising approaches include exogenous IAA application to *H. ovalis* to confirm growth promotion, co-cultivation of *H. ovalis* with the fungus in a controlled mesocosm system, and identification of auxin signaling genes of *H. ovalis*. In situ manipulative experiments comparing seagrass health and physiology (e.g., photosynthetic capacity measured by chlorophyll content) between antibiotic-treated (fungi-removed) and untreated groups would provide additional insights into the fungal role.

Improving in vitro culture protocols for local seagrasses is also a priority. Several approaches may be helpful: cultivation using a seawater-based medium [62], direct cultivation using sterilized seeds, or milder sterilization of seagrass explants using antibiotics.

## Conclusion

This study provides the first mechanistic insight into the symbiosis between seagrass and Lulworthiaceae fungi, a representative example of the plant-fungal partnership in the marine environment.

We demonstrated the ability of the Lulworthiaceae fungus *H. hongkongensis* to produce the phytohormone IAA, and elucidated its IAA biosynthesis pathways and responsible genes, including the IPA, TAM, and IAM pathways, as well as a potential flavin monooxygenase-catalyzed pathway, thereby revealing these traits for the first time in this group of cryptic marine fungi. Furthermore, *H. hongkongensis* upregulates its IAA biosynthesis genes in response to its seagrass host, most notably resulting in a significant upregulation of the IPA pathway gene. The promoting effects of *H. hongkongensis* on the model plant *A. thaliana*, which are strongly linked to its IAA-producing ability, confirm its role as a plant growth-promoting fungus. This function is likely extendable to its native seagrass host, given that IAA/auxin has been reported to positively affect seagrass growth and physiology [69–71]. Future mesocosm transplant and field manipulative experiments would be beneficial to further clarify the fungal function in detail. Additionally, assessments of the influence of fungal metabolites on *A. thaliana* growth may consider direct co-cultivation or testing using redissolved crude extract from the fungal broth [7].

Having focused on IAA above, we also examined the whole transcriptome to gain a broader perspective. Enrichment analysis of upregulated genes revealed three key pathways in this symbiosis: tryptophan metabolism (encompassing IAA biosynthesis), starch and sucrose metabolism (indicating utilization of host-derived carbohydrates), and DNA replication (reflecting adaptation and rapid growth).

Taken together, our findings indicate a beneficial symbiotic relationship in which the fungal partner *H. hongkongensis* facilitates the host seagrass growth/development by activating its IAA biosynthesis genes and secreting IAA, with concurrent enrichment of the DNA replication pathway for colonization and utilization of host starch/sucrose for energy, thereby securing its niche within *H. ovalis* tissues. This study delivers the first verification of IAA production and plant growth promotion by a seagrass-associated fungus. The findings significantly advance our understanding of the plant-fungal interactions in the marine environment.

## Supplementary Information

Below is the link to the electronic supplementary material.

Supplementary Material 1

## Data Availability

The RNA-seq data of *H. hongkongensis *have been deposited in the National Center for Biotechnology Information (NCBI) under the BioProject PRJNA1369159. The metatranscriptomic (metaT) sequencing data of seagrass below-ground tissues and rhizosphere sediments have been deposited in NCBI under the BioProject PRJNA1369154 and PRJNA1334536.
