# Supplementary material for: Insights into the Mechanism Underlying the Symbiosis between Seagrass and a Lulworthiaceae Fungus
Source: Microb Ecol. 2026 Jul 10;89:187. doi: 10.1007/s00248-026-02808-0 (PMC13658714; doi:10.1007/s00248-026-02808-0)
Supplement: Supplementary file 1 [file 248_2026_2808_MOESM1_ESM.zip › Supplementary files/Fig. S4.pdf]

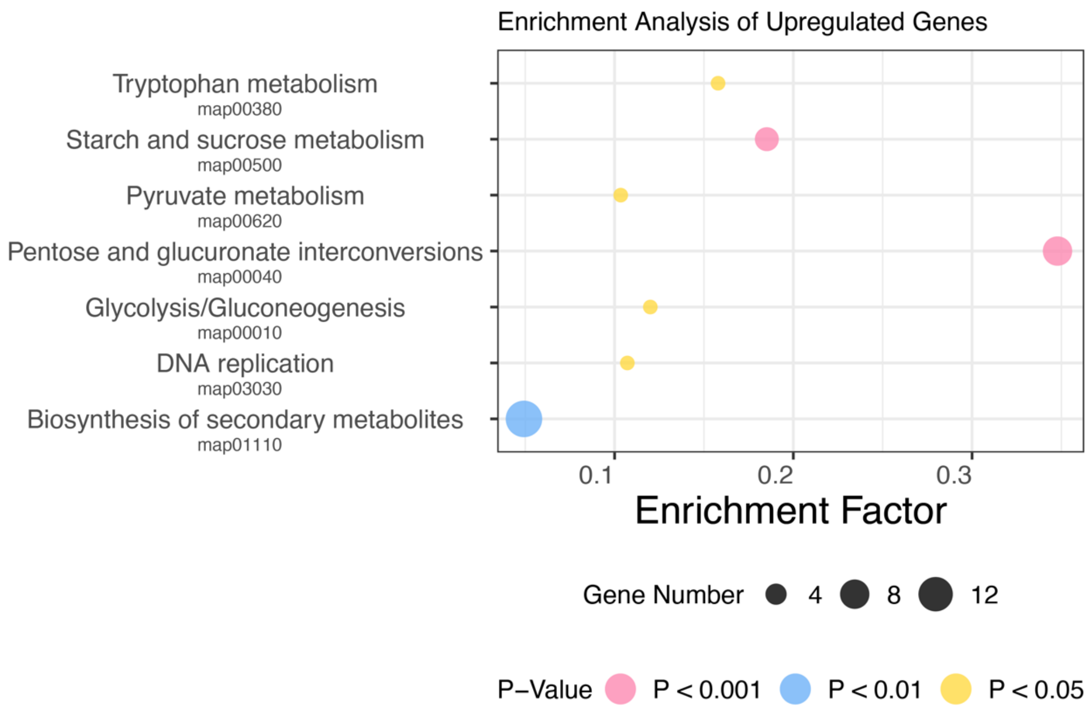

Fig. S4 Bubble plot from KEGG (Kyoto Encyclopedia of Genes and Genomes) enrichment analysis of significantly upregulated genes of *Halophilomyces hongkongensis* in seagrass *Halophila ovalis* below-ground tissues compared to rhizosphere sediments. The bubble size and color correspond to the number of genes and the *P*-value for each KEGG pathway, respectively. Only pathways with a *P*-value < 0.05 and a gene number > 2 are regarded as significantly enriched pathways and displayed.
