# Supplementary material for: Insights into the Mechanism Underlying the Symbiosis between Seagrass and a Lulworthiaceae Fungus
Source: Microb Ecol. 2026 Jul 10;89:187. doi: 10.1007/s00248-026-02808-0 (PMC13658714; doi:10.1007/s00248-026-02808-0)
Supplement: Supplementary file 1 [file 248_2026_2808_MOESM1_ESM.zip › Supplementary files/Table S1.pdf]

Table S1 Proportion of reads from RNA sequencing (RNA-seq) or metatranscriptomic (metaT) sequencing annotated as *Halophilomyces hongkongensis*

Sample Type	Sample ID	Proportion
Fungal culture (RNA-seq)	Culture1	96.43%
	Culture2	95.65%
Seagrass below-ground tissues (metaT)	Seagrass1	0.98%
	Seagrass2	1.73%
	Seagrass3	2.62%
	Seagrass4	1.43%
	Seagrass5	1.28%
	Seagrass6	1.31%
	Seagrass7	6.31%
	Seagrass8	6.22%
	Seagrass9	6.10%
	Seagrass10	6.43%
Seagrass rhizosphere sediment (metaT)	Rhizosphere1	0.31%
	Rhizosphere2	0.33%
	Rhizosphere3	0.12%
