# Supplementary material for: Insights into the Mechanism Underlying the Symbiosis between Seagrass and a Lulworthiaceae Fungus
Source: Microb Ecol. 2026 Jul 10;89:187. doi: 10.1007/s00248-026-02808-0 (PMC13658714; doi:10.1007/s00248-026-02808-0)
Supplement: Supplementary file 1 [file 248_2026_2808_MOESM1_ESM.zip › Supplementary files/Fig. S3.pdf]

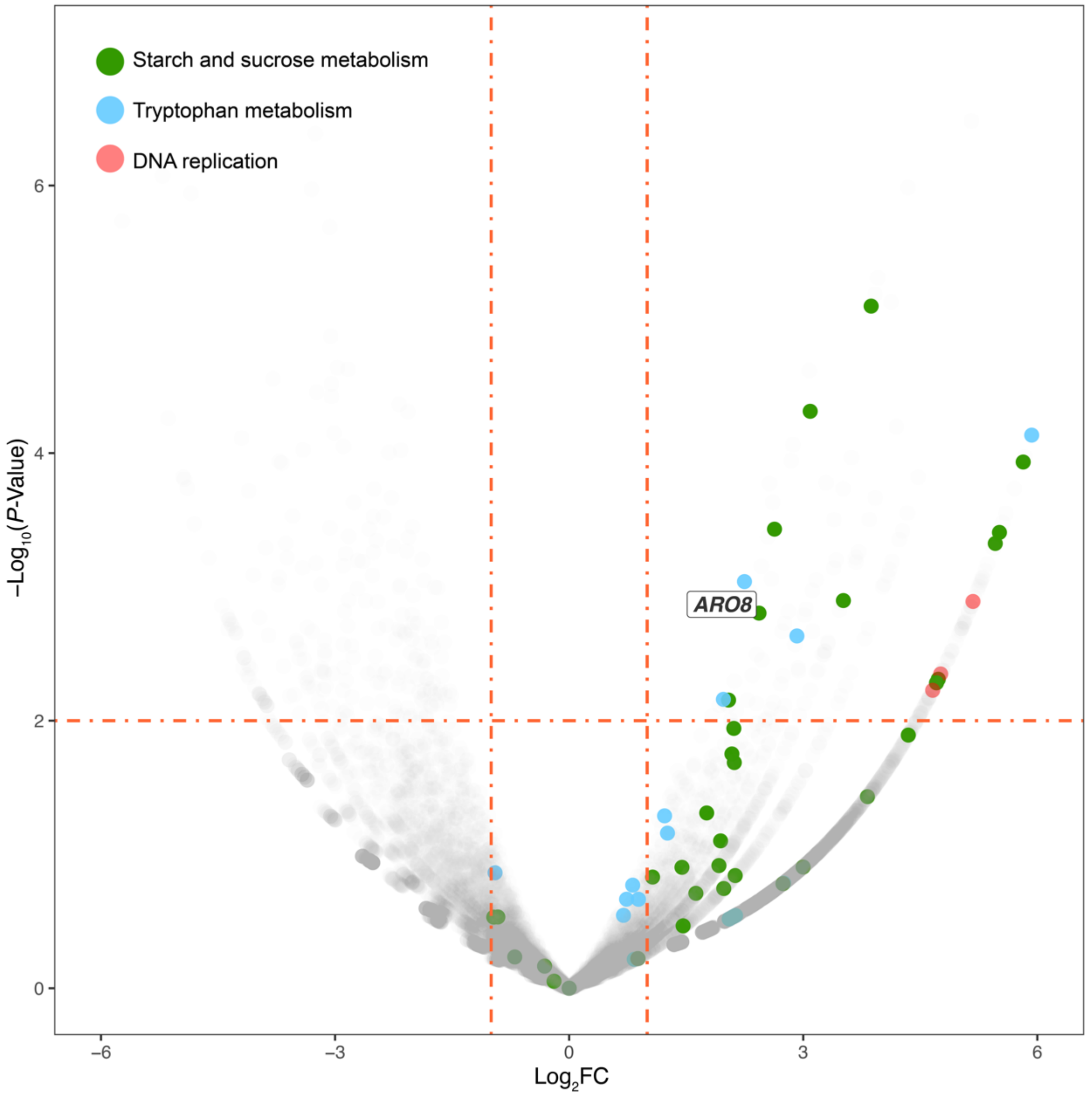

Fig. S3 Volcano plot of differentially expressed genes (DEGs) of *Halophilomyces hongkongensis* in seagrass *Halophila ovalis* below-ground tissues compared to rhizosphere sediments. DEGs involved in tryptophan metabolism (blue), starch and sucrose metabolism (green), and DNA replication (red) are highlighted. The gene *ARO8*, which plays a key role in *H. hongkongensis* indole-3-acetic acid biosynthesis, was labelled in the plot.
