# Supplementary material for: Insights into the Mechanism Underlying the Symbiosis between Seagrass and a Lulworthiaceae Fungus
Source: Microb Ecol. 2026 Jul 10;89:187. doi: 10.1007/s00248-026-02808-0 (PMC13658714; doi:10.1007/s00248-026-02808-0)
Supplement: Supplementary file 1 [file 248_2026_2808_MOESM1_ESM.zip › Supplementary files/Fig. S2.pdf]

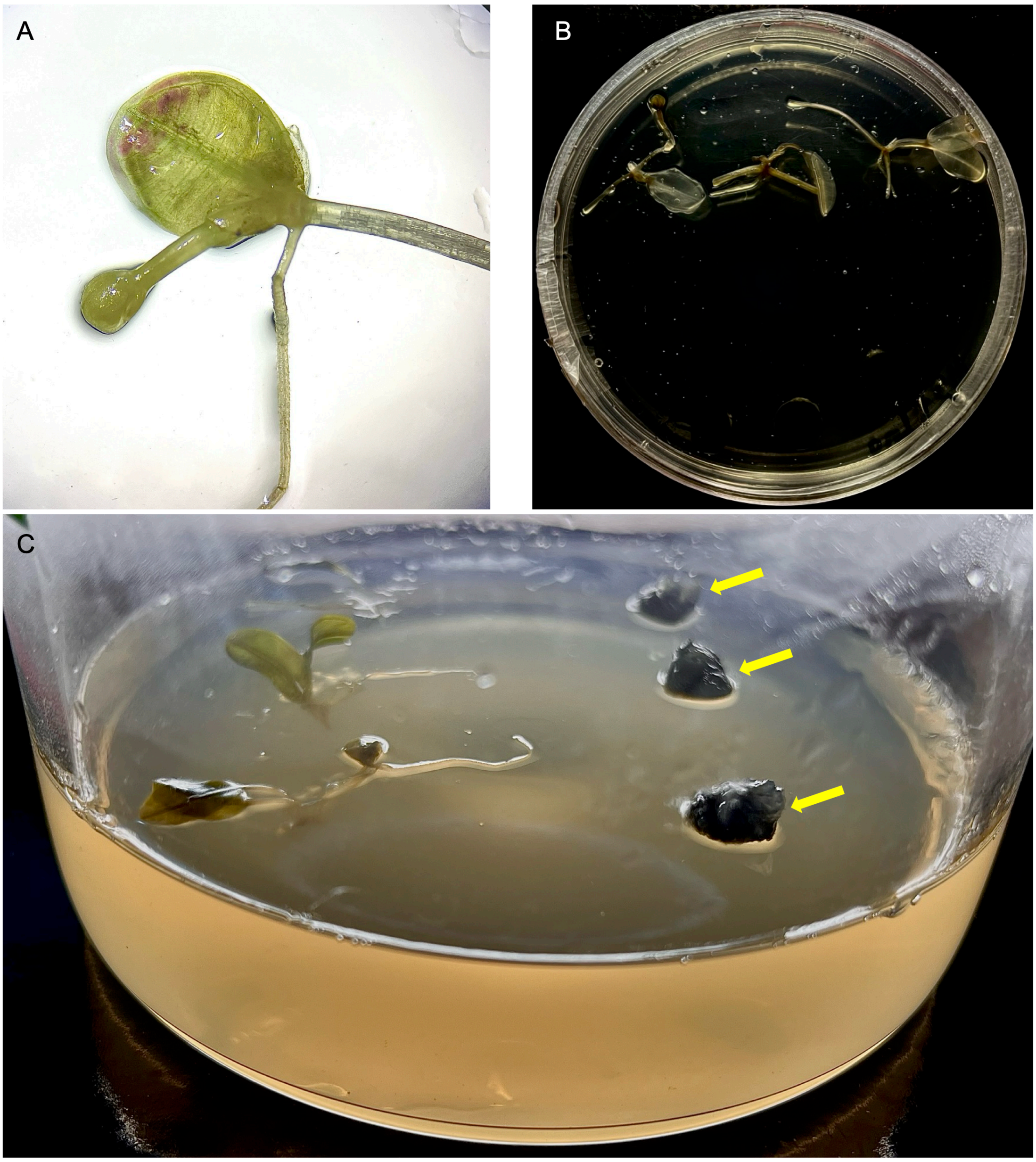

Fig. S2 The explants of the seagrass *Halophila ovalis*. (**A**) Partial *H. ovalis* containing a growing tip. (**B**) Sterilized *H. ovalis* explants on full-strength Murashige and Skoog (MS) medium in a Petri dish. (**C**) Sterilized *H. ovalis* explants co-cultured with *Halophilomyces hongkongensis* on full-strength MS medium in a culture bottle. Yellow arrow indicates the fungal inoculum.
