# Supplementary material for: Insights into the Mechanism Underlying the Symbiosis between Seagrass and a Lulworthiaceae Fungus
Source: Microb Ecol. 2026 Jul 10;89:187. doi: 10.1007/s00248-026-02808-0 (PMC13658714; doi:10.1007/s00248-026-02808-0)
Supplement: Supplementary file 1 [file 248_2026_2808_MOESM1_ESM.zip › Supplementary files/Fig. S1.pdf]

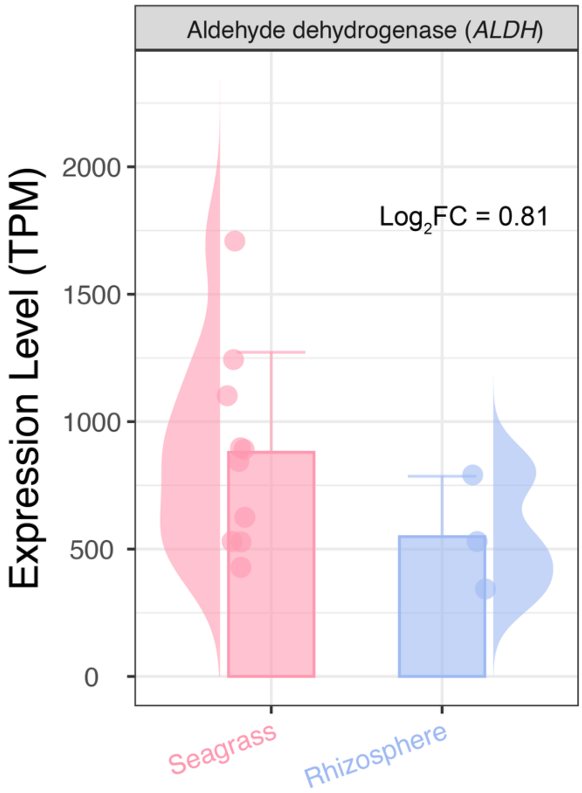

Fig. S1 Raincloud plot (combined violin plot, dot plot, and box plot) for comparison of the expression levels of *ALDH* (one of the five *H. hongkongensis* IAA biosynthesis genes) between seagrass tissues and rhizosphere sediments. The Log_2(Fold change) (Log_2FC) value from differential expression analysis using the edgeR R package is shown in the plot. The difference was not significant.
